# Complications and re-revisions after revisions of 528 metal-on-metal hips because of adverse reaction to metal debris

**DOI:** 10.1080/17453674.2020.1748351

**Published:** 2020-04-14

**Authors:** Olli Lainiala, Aleksi Reito, Jyrki Nieminen, Antti Eskelinen

**Affiliations:** Coxa Hospital for Joint Replacement and Faculty of Medicine and Health Technologies, Tampere University, Tampere, Finland

## Abstract

Background and purpose — There is limited amount of evidence about optimal revision indications, technique, and implants when performing revision surgery for metal-on-metal (MoM) hip replacements due to adverse reaction to metal debris (ARMD). We assessed which factors are related to re-revisions and complications after a revision of MoM hip arthroplasty because of ARMD. We also aimed to provide information on optimal implants for these revisions.

Patients and methods — 420 MoM total hip arthroplasties (THA) and 108 MoM hip resurfacings were implanted and later revised at our institution. We used Cox regression to analyze the factors associated with re-revisions and complications after a revision for ARMD.

Results — A re-revision was performed on 27 THAs (6%) and 9 resurfacings (8%). The most common indication for re-revision was recurrent dislocation (20 hips, 4%). Complications not leading to re-revision were seen in 21 THAs (5%) and 6 resurfacings (6%). The most common complication was dislocation treated with closed reduction in 13 hips (2%). Use of revision head size > 36mm was associated with decreased risk for dislocations. Presence of pseudotumor, pseudotumor grade, pseudotumor size, or the choice of bearing couple were not observed to affect the risk for re-revision. Non-linear association was observed between preoperative cobalt and risk for re-revision.

Interpretation — As dislocation was the most frequent post-revision complication, large head sizes should be used in revisions. Because size or type of pseudotumor were not associated with risk of re-revision, clinicians may have to reconsider, how much weight is put on the imaging findings when deciding whether or not to revise. In our data blood cobalt was associated with risk for re-revision, but this finding needs further assessment.

Adverse reaction to metal debris (ARMD) is the most common reason for failure of metal-on-metal (MoM) hip replacements (Australian Orthopaedic Association [AOA] 2018, National Joint Registry [NJR] 2018). Despite the large number of revisions, there is no consensus when a MoM hip should be revised for ARMD, how extensively debridement should be done and which implants to use (Matharu et al. 2018a). In the initial studies describing the revisions of MoM hips for ARMD the re-revision and complication rates of MoM hip revisions were high, especially in those revised for pseudotumors (Grammatopoulos et al. 2009, de Steiger et al. 2010). This led to recommending early revisions for ARMD to prevent additional tissue damage (Haddad et al. 2011, Medicines and Healthcare products Regulatory Agency [MHRA] 2012, Hannemann et al. 2013). Recently a National Joint Registry (NJR) based study reported increased risk of revision for high BMI, head and liner only revision, ceramic-on-ceramic (CoC) bearing surface, and acetabular bone grafting (Matharu et al. 2017b). Many of the factors associated with complications and re-revision after ARMD revisions, such as blood metal ion levels and cross-sectional imaging, cannot be analyzed from registry data, and studies about these have been called out by a recent review article (Matharu et al. 2018a).

Several guidelines exist concerning criteria for consideration of MoM hip revisions (Hannemann et al. 2013, MHRA 2017, US Food and Drug Administration [FDA] 2019). Most guidelines advise against revising those with normal imaging findings, low blood metal ion levels, and minor symptoms, and suggest considering revisions in those with major symptoms, large soft tissue lesions, and extremely high blood metal ion levels. Between these extremities falls the “gray area of ARMD,” for which cases the guidelines suggest individual evaluation. We aimed to provide information to help decision-making with these patients.

In this study, we report the reasons for re-revision and complications after revisions of MoM hip due to ARMD. We also analyze the risk factors for re-revision and complications. We hypothesized that large solid/mixed type pseudotumors and high blood metal ion levels would be associated with poor revision results, and that use of large head size would decrease the risk for post-revision complications. We also were interested in whether some bearing surface(s) performed better than others, or whether there is a difference between liner-only and cup revision, as those are variables that surgeons can choose.

## Patients and methods

We searched all the patients with primary MoM hip replacement implanted and later revised due to ARMD at our institution. We included all patients who were revised a minimum of 1 year before the data collection in September 2018 and had pre-revision cobalt (Co) and chromium (Cr) measurements and imaging available for the diagnosis of ARMD. The primary surgery (October 2001–August 2011) as well as revision surgery (September 2010–September 2017) of all the patients included was performed at our high-volume center. No referral patients were included.

After the recall of Articular Surface Replacement (ASR, Depuy Orthopaedics, Warsaw, IN, USA) (Depuy Orthopaedics 2010), we established an intensified screening program for all ASR MoM hip replacements at our institution. Screening included whole blood Co and Cr measurements, physical examination and cross-sectional imaging with MRI as primary imaging modality. Initially, ultrasound was used only if there were contraindications for MRI, but later the use of ultrasound was increased due to lower cost. If the patient had progressive symptoms or elevating whole-blood metal ion levels, imaging was repeated. MoM hip replacement brands other than ASR were also included in the screening from 2012, but cross-sectional imaging was performed only for the patients with symptoms or elevating whole-blood Co or Cr levels.

Whole-blood Co and Cr measurements were performed with dynamic reaction cell inductively coupled plasma mass spectrometry (Agilent 7500cx, Agilent Technologies, Santa Clara, CA, USA). Preoperative MRIs were performed with Siemens Magnetom Avanto 1.5 T (Siemens Healthcare, Erlangen, Germany) or GE Signa HD 1.5 T (General Electric Healthcare, Waukesha, WI, USA) and ultrasound examinations were performed with Logiq E9 (GE Healthcare, USA). Imaging findings were graded according to a previously described system by musculoskeletal radiologists (Matthies et al. 2012).

Revision surgery for ARMD was considered if (1) a thick-walled pseudotumor with atypical contents or solid-type pseudotumor was seen in imaging, or the patient had (2) elevated whole blood Co or Cr levels and hip symptoms despite normal imaging finding, or (3) continuously symptomatic hip or progressive symptoms regardless of normal imaging findings or whole blood metal concentrations, or (4) the patient had progressively increasing whole-blood metal ion levels, even without symptoms or findings in cross-sectional imaging. Co and Cr were considered as elevated if they exceeded 5 µg/L (Hart et al. 2011). The diagnosis of ARMD was based on intraoperative findings irrespective of preoperative working diagnosis. Failure was classified as being due to ARMD if metallosis was present or there was macroscopic synovitis in the joint, and/or a pseudotumor was found during revision and perioperatively there was no evidence of component loosening or periprosthetic fracture. Infection was ruled out by at least 5 bacterial cultures obtained during revision surgery. Histopathological samples were collected during revision to further support the validity of the intraoperative diagnosis.

Revision was defined as surgery including a change of at least 1 component (stem, head, liner, or/and cup). In revision surgery of THA, if the stem was well fixed and correctly positioned it was retained and only the cup or liner revised. In resurfacing revisions, the femoral neck was cut and a stem implanted. The revision implants were chosen based on the surgeon’s preference. If a ceramic head was used, a titanium sleeve adapter was applied, even if there were no signs of corrosion in the taper. In 8 revisions with an unusually large pseudotumor extending into the intrapelvic region, additional resection of the intrapelvic pseudotumor was performed through an ilioinguinal approach to complement resection from posterior approach.

After the revision, anteroposterior and lateral plain radiographs of the hip and anteroposterior pelvic radiographs were obtained, blood Co and Cr measurements were performed at 2, 6, and 12 months, and the patient was clinically evaluated by an orthopedic surgeon at 2 and 12 months, and thereafter at 2-year intervals. Acetabular inclination was measured from anteroposterior plain radiographs using ischial tuberosities as reference, and anteversion was measured from cross-table radiographs using the horizontal plane as a reference.  

### Statistics

Means (SD) are presented for normally distributed variables, and medians (ranges) for variables with non-Gaussian distribution. In Kaplan–Meier survival analysis the results are reported till the year with at least 20 hips at risk. A Cox regression model was used to analyze factors associated with re-revision, and proportional hazards assumption was analyzed using Schoenfeld’s residuals. No violation of proportional hazards assumption was met. Directed acyclic graphs (DAG) were used to guide the selection of variables for the model (Shrier and Platt 2008). As we had several variables of interest (revision head size, bearing surface, pseudotumor, type of revision, and pre-revision Co), we created DAGs for each variable to ensure appropriateness of multivariable analysis. Co level was analyzed as a linear variable, but also nonlinear relationships for Co were investigated fitting restricted cubic splines. Cox regression analysis was done by including pre-revision Co level and appropriate covariates based on DAG using restricted cubic spline with 4 knots. HRs were plotted using median Co value as reference and subtracting the constant so that the width of the 95% confidence interval (CI) was zero for reference value. An ANOVA test was used to assess the statistical significance of nonlinearity. IBM SPSS Statistics version 25 (IBM Corp, Armonk, NY, USA) and R v3.2.1 (R Foundation for Statistical Computing, Vienna, Austria) were used for statistical analyses.

### Ethics, funding, and potential conflicts of interest

This study was approved by the local ethics committee (registration IDs R11006 and R11195). This work was supported by the competitive research funds of Pirkanmaa Hospital District, Tampere, Finland, representing governmental funding. Individual potential conflicts of interests: OL: none. AR: Orion LTD, paid lecture. JN: none. AE: Zimmer Biomet, paid lectures; Depuy Synthes and Zimmer Biomet, institutional research support (not related to current study). 

## Results

**Figure 1.**
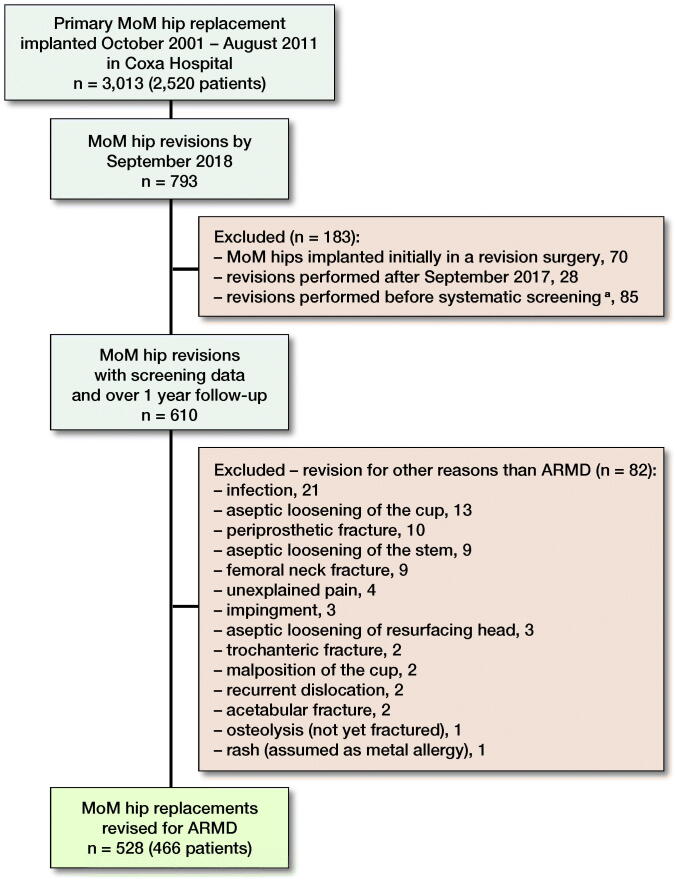
Flow chart of patient inclusion. MoM = metal-on-metal; Co = cobalt; Cr = chromium; ARMD = adverse reaction to metal debris. **^a^**No preoperative imaging or Co/Cr measurements.

3,013 MoM hips in 2,520 patients were identified. By September 2018, 793 revisions of MoM hip replacements had been performed at our institution. As we included only hips revised for ARMD without any other indications for revision, 528 MoM hips in 466 patients were included in this study (Figure 1). 420 (80%) of the implants used in primary surgery of the revised hips were stemmed MoM total hip arthroplasties (THA) and 108 (20%) were hip resurfacings (Table 1).

**Table 1.** Patients’ pre-revision characteristics

	Total hip arthroplasty	Hip resurfacing
Factor	n = 420	n = 108
Women, n (%)	230 (55)	72 (67)
Age at revision, mean (SD)	67 (8.5)	57 (9.7)
Pre-revision Co, µg/L		
median (range)	11.8 (0.2–192)	11.8 (0.4–225)
(IQR)	(7.0–20)	(2.1–36)
Pre-revision Cr, µg/L		
median (range)	7.9 (0.4–156)	7.4 (0.8–125)
(IQR)	(2.3–6.5)	(2.2–20)
Any symptoms n (%)	272 (65)	79 (73)
Imaged with		
MRI, n (%)	301 (72)	83 (77)
ultrasound, n (%)	119 (28)	25 (23)
Head size, median mm (range)	47 (28–59)	49 (42–63)
Years between primary and		
revision surgery, mean (SD)	6.3 (2.4)	6.7 (2.4)
BMI, mean (SD)	28 (4.7)	28 (4.5)
ASA class at primary		
1/2/3/4/NA	40/219/144/3/14	42/51/10/2/3

SD = standard deviation; IQR = interquartile range; NA = not available.

Implants used in primary surgeries are listed in Table 2 and implants used in revision surgeries in Table 3 (Supplementary data). Pre-revision cross-sectional imaging and whole blood Co and Cr measurements were available for all patients included in the study. Median time between the pre-revision imaging and revision surgery was 4.3 months (5 days–24 months). The pre-revision imaging findings are listed in Table 4. The median time between pre-revision Co and Cr measurements and revision was 4.8 months (2 days–19 months). For patients with stemmed MoM THAs, there were 374 cup revisions, 42 liner-only revisions, 1 revision with dual mobility cup system, and in 3 patients with stemmed MoM THA the stem component was also changed (2 cup revisions and 1 liner revision). In all 108 resurfacing revisions, the cup was changed and a stem implanted. Median head size of the revision components was 36 mm (28–48). Median acetabular inclination of the cups implanted at revision was 45° (SD 7) and acetabular anteversion 27° (SD 8).

**Table 4.** Pre-revision imaging findings with MRI or ultrasound

	Total hip arthroplasty		Hip resurfacing	
Grade	n (%)	Size, cm median (range)	n (%)	Size, cm median (range)
0	203 (48)		50 (46)	
1	82 (20)	5.7 (1.2–25)	22 (20)	4.9 (2.4–10)
2A	48 (11)	6.4 (3.0–17)	8 (7)	7.5 (4.3–11)
2B	68 (16)	8.4 (3.6–30)	25 (23)	7.9 (2.5–19)
3	19 (5)	6.0 (2.4–13)	3 (3)	10.7 (5.4–13)
Total	420		108	

Imaging findings are classified according to a previously described grading system (Matthies et al. 2012). Grade 0 represents normal imaging finding; grade 1 represents thin-walled fluid-filled pseudotumor; grade 2A a fluid-filled pseudotumor with thick or irregular walls; 2B a pseudotumor with atypical contents; and grade 3 a predominantly solid pseudotumor.

**Figure 2.**
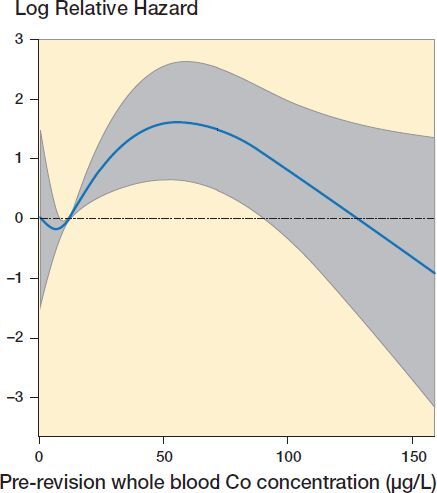
Non-linear association was seen between pre-revision blood cobalt (Co) concentration and risk for revision. Preoperative Co between approximately 20 and 90 µg/L was associated with increased risk for re-revision. Hazard ratios are presented in relation to median Co of 11.8 µg/L. Gray area represents 95% confidence interval.

A re-revision requiring a change of any component was performed on 36 hips (36 patients, 27 involving THA [6%], 9 involving resurfacings [8%]). Median time from revision to re-revision was 4.6 months (7 days–7.3 years). There were complications not leading to change of the components involving 27 hips (21 THA [5%], 6 resurfacings [6%]). The median time from revision to complication was 4.8 months (47 days–7.6 years). The reasons for re-revisions and complications are listed in Table 5. Among the group of patients with complications not leading to revision and the re-revision group there was overlapping in 3 cases. Therefore, 60 (11%) hips experienced at least 1 complication that was or was not treated with revision surgery. The median post-revision follow-up time for those in follow-up without revision was 5.2 years (1.3–8.0). 21 hips (20 patients) were lost to follow-up as they either moved outside our hospital district, refused the follow-up or could not be contacted after mean follow-up of 2.7 years (1.1–5.0); all were included in the analyses until the last contact. 12 patients (12 hips) died during the study period (median follow-up 3.8 years [0.2–6.3]) for reasons not related to the prosthesis. The 7-year implant survivorship after revisions of stemmed MoM THA was 94% (CI 91–96) and after revisions of MoM resurfacing 91% (CI 86–97). In Cox regression analysis, none of the variables tested were observed to have statistically significant association with re-revisions (Table 6, Supplementary data). When Co was treated as a non-linear variable, we observed a non-linear relationship in which pre-revision Co concentration between 20 and 90 µg/L was associated with increased risk for re-revision (Figure 2).

**Table 5.** Indications for re-revisions and complications after the revisions of metal-on-metal total hip arthroplasty (THA) and resurfacing arthroplasties

	THA	Resurfacing
after closed reduction	18	2
**Re-revision indication**		
Mechanical reasons		
Recurrent dislocation or instability		
Periprosthetic femoral fracture	1	3
Acetabular fracture	2	0
Fracture of stem component	0	1
Aseptic loosening of stem	1	1
Non-mechanical reasons		
Recurrent ARMD	1	1
Infection	4	1
Total	27	9
**Complication**		
Mechanical complication		
Dislocation(s) treated with		
closed reduction	11	2
Acetabular fracture ^a^	3	0
Trochanteric fracture ^b^	1	0
Fascial rupture ^c^	2	1
Hernia	1	0
Non-mechanical complication		
Residual pseudotumor	0	2
Pulmonary embolism	1	0
Deep vein thrombosis	1	1
Bowel occlusion	1	0
Total	21	6

ARMD = adverse reaction to metal debris;

**^a^**non-operative treatment;

**^b^**operated with titanium plate without change of components;

**^c^**re-suture without change of components.

As dislocation was the most common reason for re-revision as well as the most common complication, a separate analysis of risk factors for instability was performed. The instability group included those suffering a dislocation not leading to re-revision (13 hips) and those who underwent a re-revision due to recurrent dislocations/instability (20 hips). Thus 33 hips were included in the instability group. In both univariable and multivariable analyses revision head size of 36 mm or smaller was associated with increased risk for dislocation (Table 7, Supplementary data).

As our study included patients with bilateral revisions, analyses in Tables 6 and 7 were re-performed with exclusion of left hips of bilateral patients to rule out potential bias by clustered observations. This did not change the results (analyses not shown). 

## Discussion

A large number of revisions have been performed on MoM hip replacements due to ARMD all over the world (NJR 2018, AOA 2018); however, there is still a limited amount of information regarding on which patients the revision should be performed. We aimed to contribute to the understanding concerning revisions of MoM hips due to ARMD. A strength of our study is our unselected patient group, as our hospital is a primary center and all the primary surgeries and revisions were performed at our institution. Therefore, referral patients do not cause selection bias in this study. The revision threshold may have been lower in our institution compared with some other institutions, and our previously reported survivals are lower compared with implant registries, possibly due to higher proportion of ASR MoM hip replacements (Lainiala et al. 2019), which should be accounted for when comparing these results with results from other centers.

Our study has some limitations. 1st, as this is retrospective study and patients from several surgeons were included, there might have been variation in the threshold for revision surgery and the surgical technique (for example the extent of tissue resection and choice of implants used). Further, there were several brands of stemmed MoM THAs and MoM hip resurfacings as well as revision implant brands used. For studying the clinical significance of preoperative factors, the use of a single revision implant brand would have been the best option. On the other hand, using similar numbers of metal-on-polyethylene (MoP), ceramic-on-ceramic (CoC), and ceramic-on-polyethylene (CoP) bearing surfaces with a single cup brand in a randomized setting would have allowed us to compare the performance of the different revision bearing surfaces. 2nd, the amount of re-revisions and complications is only 1 perspective. Oxford Hip Scores have also been registered at our institution, but we decided to concentrate on the objective endpoints in this study, and the patient-reported outcome measures will be reported in future.

We observed re-revisions requiring change of any component in 36 hips (7%) and a complication not requiring change of components in 27 (5%) hips. The 7-year implant survivorship of 94% after revision of stemmed THAs and 91% after revision of hip resurfacings is better than the overall 7-year re-revision rate of 14.2% for all bearing surfaces and indications in the NJR registry (NJR 2018). 1 of the earliest hip resurfacing cohorts that raised concerns about the complications after ARMD revisions has reached follow-up of 10 years and a re-revision rate of 38% was reported (Grammatopoulos et al. 2009, Matharu et al. 2017c). Poor early results led several authors to recommend early intervention (Grammatopoulos et al. 2009, De Smet et al. 2011, Su and Su 2013). Since then, re-revision rates between 0% and 4% and complication rates between 0% and 10% have been reported for revisions of MoM hip resurfacings (Eswaramoorthy et al. 2009, De Smet et al. 2011, Gross and Liu 2014), and 8-22% re-revision rates and 8-38% complication rates for MoM THAs (Munro et al. 2014, Stryker et al. 2015, Wyles et al. 2014, van Lingen et al. 2015, Jennings et al. 2019). Many cohorts have described dislocation as a common problem after a revision of MoM hip replacement (Grammatopoulos et al. 2009, De Smet et al. 2011, Munro et al. 2014, Stryker et al. 2014, van Lingen et al. 2015, Jennings et al. 2019). Despite the early warnings about poor results of ARMD revisions, a recent study from the NJR registry showed that the re-revision rates were lower in MoM hips revised for ARMD compared with those revised for non-ARMD (Matharu et al. 2018b).

A larger head size has been suggested to decrease the risk for poor outcome (Matharu et al. 2019), which is in line with our result: head sizes larger than 36 mm decreased the risk for dislocation. A recent study reported solid pseudotumors with abductor deficiency to be associated with post-revision complications (Liow et al. 2016), but neither our study nor a study by Matharu et al. (2019) found evidence of an association between cross-sectional imaging findings and revision results. Guidelines have put weight on the type and the size of the soft tissue abnormalities when considering revision (Hannemann et al. 2013, MHRA 2017, FDA 2019), but it seems that mixed or solid-type pseudotumors do not necessarily cause a high risk of complications after revision. Of course, pseudotumors are not the only type of lesions related to ARMD and muscle deficiency and osteolysis need to be considered. A CoC bearing surface is reported to be associated with risk for poor outcome in 2 recent British studies (Matharu et al. 2017b, 2019), but neither our study nor an Australian registry-based study (Wong et al. 2015) found a difference between different bearing surfaces used for MoM revisions. Ceramic heads are used at our institution to minimize metal release from the trunnion–taper junction, and nowadays our bearing surface of choice is CoP, as use of CoC is associated with occasional squeaking (McDonnell et al. 2013, Salo et al. 2017). Using CoP bearings with head size > 36 mm may lead to a very thin polyethylene liner, and we certainly try to avoid this—especially if the patient is young and active. Currently, our policy is to use a CoP bearing mainly with a 36 mm head. In patients with a very large cup size that allows usage of > 36 mm heads with adequate thickness of the polyethylene liner, > 36 mm heads can be considered. However, if satisfactory stability cannot be achieved with the CoP bearing, then we would choose either a constrained liner or dual mobility bearing.

Jennings et al. (2019) reported higher median Co and Cr for patients with post-revision complications compared with those without complications. In our study, the association was non-linear, and only Co 20–90 µg/L was associated with an increased risk for re-revision. A few recent studies observed no association with preoperative metal ion levels and poor outcome (Liow et al. 2016, Matharu et al. 2019). The possible association between whole-blood metal ion concentrations and revision results is clearly complex, needs further investigation and no single metal ion value can be given as a threshold for revision. An explanation as to why higher Co values were not associated with increased risk might be that extremely high Co levels have led to revision with lesser imaging findings and symptoms compared with only slightly or moderately elevated whole blood Co levels. Recent studies (Matharu et al. 2017a, 2017b, 2019) observed increased risk for poor outcome in patients with selective component revision (some of the components retained). We did not observe a difference between THAs treated with head and liner exchange, and those with the cup revised. Therefore, we still consider head and liner revision to be a viable option in a subset of patients with a well-fixed and positioned modular cup.

## Conclusion

Dislocation is the most frequent post-revision complication after ARMD revisions and using larger head sizes than 36 mm decreases the risk for dislocation. Neither the size nor the grade of pseudotumor were associated with the outcome of revision, but this should be further evaluated with inclusion of other variables describing tissue damage. We recommend using a CoP bearing with as large a head size as feasible and choosing either a constrained liner or dual mobility bearing if satisfactory stability cannot be reached with the CoP bearing.  

## Supplementary Material

Supplemental Material

## Supplementary data

Tables 2, 3, 6, and 7 are available as supplementary data in the online version of this article, http://dx.doi.org/10.1080/17453674.2020.1748351

The authors would like to thank Ella Lehto RN, and Heli Kupari RN, Coxa Hospital for Joint Replacement, Tampere, Finland, for maintaining their study database. They also thank the orthopedic surgeons from Coxa Hospital who performed the surgeries analyzed in this study. They thank the musculoskeletal radiologists who performed the ultrasounds and analyzed MRI studies. The authors also thank the physiotherapists from Coxa Hospital.
