# Supplementary material for: Complications and re-revisions after revisions of 528 metal-on-metal hips because of adverse reaction to metal debris
Source: Acta Orthop. 2020 Apr 14;91(4):365–71. doi: 10.1080/17453674.2020.1748351 (PMC8023960; doi:10.1080/17453674.2020.1748351)
Supplement: Supplemental Material [file IORT_A_1748351_SM8792.pdf]

Supplementary data

Table 2. Implants used in primary MoM surgeries

Cup brand	Manufacturer	MoM THAs	Resurfacings
ASR	Depuy	237	68
BHR	Smith & Nephew	35	30
Conserve +	Wright Medical	2	
Continuum	Zimmer	2	
Durom	Zimmer	11	8
M2A	Biomet	12	
Mitch	Stryker	5	
Pinnacle	Depuy	68	
R3	Smith & Nephew	31	
ReCap	Biomet	12	2
Universal	Biomet	4	
Vision	Biomet	1	
Total		420	108

THA = total hip arthroplasty; ASR = Articular Surface Replacement; BHR = Birmingham Hip Resurfacing.
Full affiliations of manufacturers:
Depuy Orthopaedics, Warsaw, IN, USA;
Smith & Nephew, Memphis, TN, USA;
Wright Medical, Memphis, TN, USA;
Zimmer, Warsaw, IN, USA;
Biomet, Warsaw, IN, USA;
Stryker, Mahwah, NJ, USA.

Table 3. Implants used in revision surgeries

Manu-facturer	Cup brand	n	Bearing surface	n	Head size median (range)
Zimmer	Continuum	190	CoP	99	36 (36–44)
			CoC	58	40 (36–40)
			MoP	23	36 (32–40)
			Constrained	10	36 (28–36)
Depuy	Pinnacle	108	CoC	59	36 (36–36)
			CoP	34	36 (36–36)
			MoP	15	36 (36–36)
Zimmer	Trabecular Metal	105	MoP	55	36 (28–36)
			CoP	42	36 (28–36)
			Constrained	8	36 (32–36)
Depuy	Deltamotion	70	Monoblock CoC	70	48 (40–48)
Biomet	Exceed	8	CoC	6	36 (36–40)
			CoP	2	36 (36–36)
Zimmer	Regenerex	2	Constrained	2	36
Stryker	Trident	1	CoC	1	36
Total		484		484	
**Liner only revisions (old cup was retained)**					
Depuy	Pinnacle	35	CoP	25	36 (36–36)
			MoP	6	36 (36–36)
			CoC	4	36 (36–36)
	R3	8	CoP	5	36 (32–36)
			MoP	2	36 (36–36)
			CoC	1	36
Total		43		43	
**Dual mobility revision**					
Stryker	ADM	1	PoM	1	28

CoC = ceramic-on-ceramic; CoP = ceramic-on-polyethylene; MoP = metal-on-polyethylene; PoM = polyethylene-on-metal (old Birmingham Hip Resurfacing cup retained); ADM = anatomic dual mobility. Full affiliations of manufacturers: See Table 2.

Table 6. Cox regression univariable and multivariable analysis of risk factors for re-revision after revision. Values are number, Hazard ratio (95% CI)

Factor	Univariable		Multivariable	
Primary implant type				
Hip resurfacing	108	1.2 (0.6–2.6)		
THA	420	Reference		
Primary head size (mm) [a]	528	1.03 (0.97–1.09)		
Sex				
Male	226	1.3 (0.7–2.5)		
Female	302	Reference		
Age at revision (years) [a]	528	0.99 (0.96–1.02)		
Bearing surface				
CoC	199	Reference	188	Reference
CoP	207	1.6 (0.7–3.5)	193	1.3 (0.4–4)
MoP	101	1.5 (0.6–3.9)	93	1.7 (0.5–6)
Constrained	20	1.9 (0.4–8)	19	2.1 (0.3–14)
Imaging finding				
Pseudotumor	275	1.1 (0.6–2.2)	255	1.0 (0.5–2.1)
No pseudotumor	253	Reference	238	Reference
Grade of pseudotumor [b]				
0	253	Reference	238	Reference
1	104	1.2 (0.5–2.8)	94	1.0 (0.4–2.7)
2A	56	1.1 (0.4–3.3)	52	1.2 (0.4–3.8)
2B	93	1.0 (0.4–2.6)	87	0.8 (0.3–2.2)
3	22	1.4 (0.3–6)	22	1.3 (0.3–6)
Pseudotumor size (cm) [a]	275	1.03 (0.92–1.14)	255	1.00 (0.86–1.15)
Pre-revision cobalt (µg/L) [a]	528	1.00 (0.99–1.01)	493	1.00 (0.99–1.01)
Years from primary surgery to revision [a]	528	1.12 (0.98–1.29)		
Revision type				
Cup revision	374	Reference	352	Reference
Liner revision	42	0.8 (0.2–3.3)	39	1.1 (0.2–6)
Resurfacing revision	108	1.2 (0.6–2.7)	102	1.1 (0.4–3.0)
Cup and stem revision	3	Not analyzed		
Dual mobility revision	1	Not analyzed		
Revision head size (mm)				
28–32	26	2.3 (0.4–12)	21	2.2 (0.3–17)
36	363	2.5 (1.0–7)	338	2.1 (0.6–7)
38–48	139	Reference	134	Reference
Acetabular inclination (°) [a]	528	0.98 (0.93–1.03)		
Acetabular anteversion (°) [a]	528	1.0 (0.95–1.04)		
ASA class				
1	82	Reference		
2	270	1.0 (0.4–2.5)		
3	154	1.0 (0.4–2.8)		
BMI [a]	519	1.01 (0.94–1.09)		

[a] For continuous variables, hazard ratios are presented per increase of 1 unit.
[b] Grade of pseudotumor: See Table 4.
Analysis of bearing surfaces excluded one dual-mobility implant.
Class 4 of ASA grading was excluded due to including only 5 patients.
THA = total hip arthroplasty; CoC = ceramic-on-ceramic; CoP = ceramic-on-polyethylene; MoP = metal-on-polyethylene; BMI = body mass index.
Based on directed acyclic graphs (DAG, supplement) covariates included in the model for revision head size, bearing surface, and type of revision (liner/cup/resurfacing) were type of primary implant, sex, age, pseudotumor grade and size, pre-revision cobalt, primary head size, ASA class, inclination, anteversion, time from primary to revision, BMI, and the other two of bearing surface/revision head size/type of revision. In the model for pseudotumor (presence, grade, and size), type of primary implant (THA/resurfacing), primary head size, pre-revision cobalt, and time from primary to revision surgery were omitted from the model based on DAG. In analysis of pseudotumor size, those without pseudotumor were excluded (not marked as 0 cm). In analysis of pre-revision cobalt, pseudotumor was omitted based on DAG.

Table 7. Cox regression univariable and multivariable analysis of risk factors for dislocations after the revision. Values are number, Hazard ratio (95% CI)

Factor	Univariable		Multivariable	
Primary implant type				
Hip resurfacing	107	0.5 (0.2–1.5)		
THA	401	Reference		
Primary head size (mm) [a]	508	1.05 (0.99–1.12)		
Sex				
Male	218	2.2 (1.1–4)		
Female	290	Reference		
Age at revision (years) [a]	508	0.99 (0.95–1.02)		
Imaging finding				
Pseudotumor	261	1.0 (0.5–2.0)	242	1.0 (0.5–2.1)
No pseudotumor	247	Reference	236	Reference
Grade of pseudotumor [b]				
0	247	Reference	236	Reference
1	103	0.9 (0.4–2.3)	93	0.9 (0.3–2.5)
2A	55	1.1 (0.4–3.3)	51	1.4 (0.4–4.4)
2B	85	1.1 (0.4–2.8)	80	1.0 (0.4–2.8)
3	18	0.8 (0.1–6)	18	0.8 (0.1–6.2)
Pseudotumor size (cm) [a]	261	1.02 (0.94–1.10)	242	1.00 (0.83–1.21)
Pre-revision cobalt (µg/L) [a]	508	1.00 (0.99–1.01)	478	1.00 (0.99–1.01)
Years from primary surgery to revision [a]	508	1.13 (0.98–1.31)		
Revision head size (mm)				
28–32	18	9 (1.8–43)	15	10 (1.4–70)
36	351	4 (1.3–14)	328	4 (1.2–16)
38–48	139	Reference	135	Reference
Acetabular inclination (°) [a]	508	1.03 (0.98–1.08)		
Acetabular anteversion (°) [a]	508	1.01 (0.98–1.06)		
ASA class				
1	81	Reference		
2	257	1.3 (0.5–4)		
3	149	1.6 (0.5–5)		
BMI [a]	499	1.04 (0.97–1.12)		

[a] For continuous variables, hazard ratios are presented per increase of 1 unit.
[b] Grade of pseudotumor: See Table 4.
Constrained implants were excluded from this analysis as per design they do not dislocate. Class 4 of ASA grading was excluded due to including only 5 patients. THA = total hip arthroplasty. BMI = body mass index.
Based on directed acyclic graphs (DAG, supplement) covariates included in the model for revision head size were type of primary implant, sex, age, pseudotumor grade and size, pre-revision cobalt, primary head size, ASA class, inclination, anteversion, time from primary to revision, and BMI. In the model for pseudotumor (presence, grade and size), type of primary implant (THA/resurfacing), primary head size, pre-revision cobalt, and time from primary to revision were omitted from the model based on DAG. In analysis of pseudotumor size, those without pseudotumor were excluded (not marked as 0 cm). In analysis of pre-revision cobalt, pseudotumor is omitted based on DAG.

Directed acyclic graphs (DAG)

Reference: Reducing bias through directed acyclic graphs, Shrier and Platt 2008

"Interpretation: If X (exposure of interest) is dissociated from the outcome after the last step, then the statistical model chosen (i.e. one that includes only the chosen covariates) minimizes the bias of the estimate of X on the chosen outcome."

 Acta Orthopaedica 2020; 91 (DOI: 10.1080/17453674.2020.1748351)

1st DAG (revision head size)
- X (exposure of interest – revision head size
- Outcome – re-revision

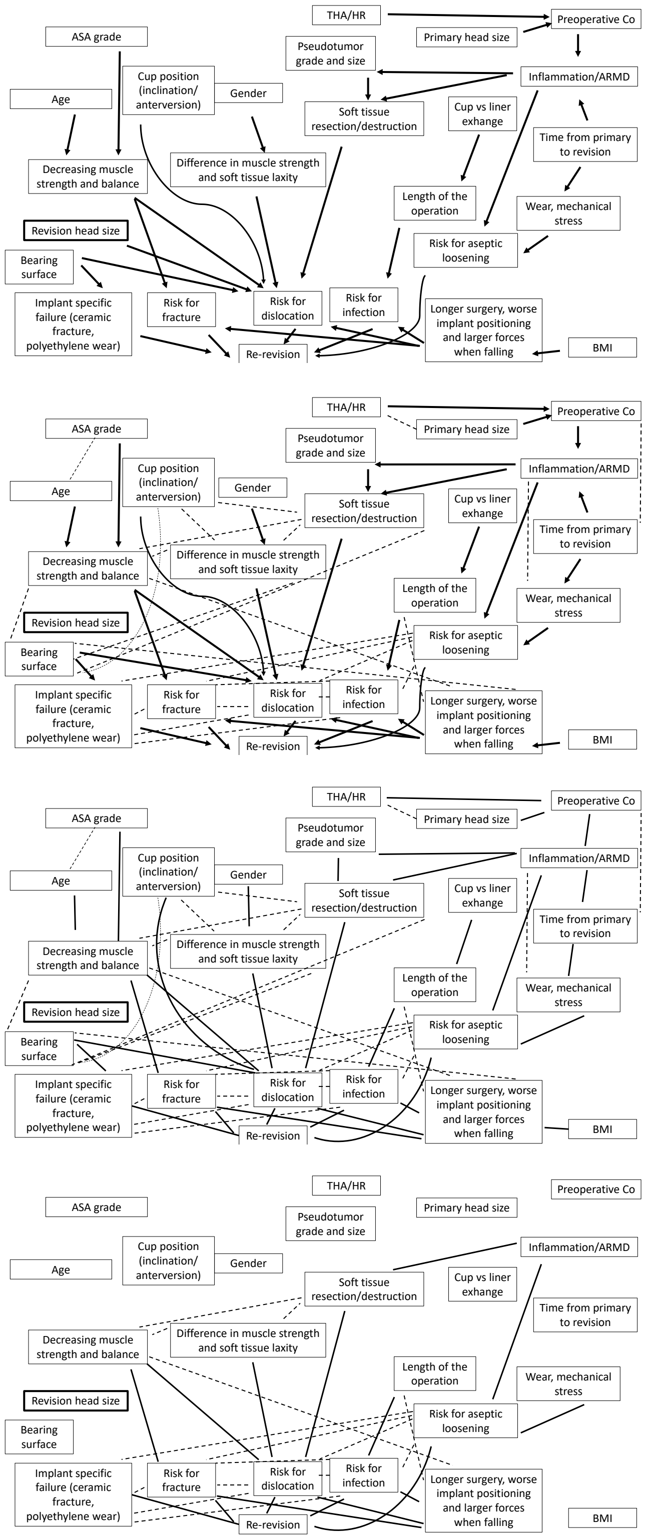

1st DAG (revision head size)
- X (revision head size) is dissociated from the outcome – all chosen covariates included

2nd DAG (revision bearing surface)
- X (exposure of interest) – bearing surface
- Outcome – re-revision

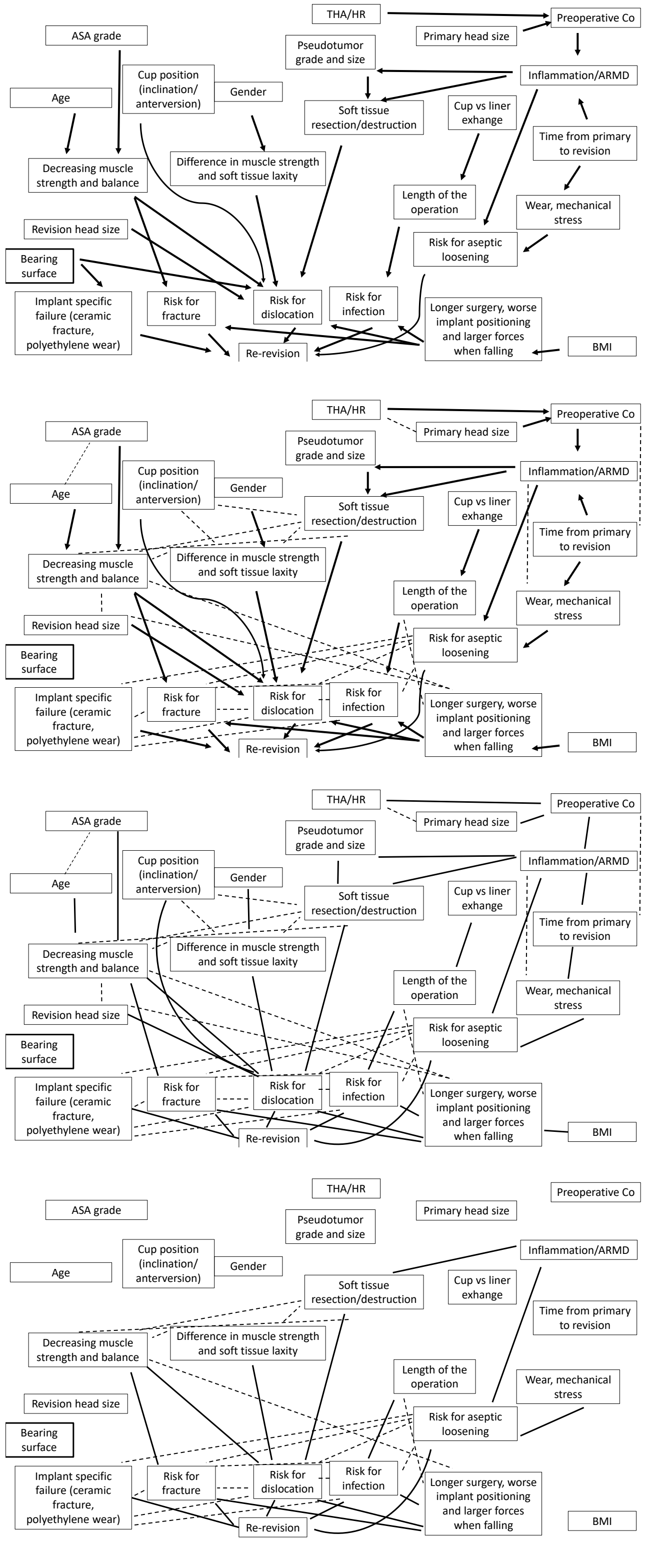

2nd DAG (revision bearing surface)
- X (bearing surface) is dissociated from the outcome – all chosen covariates included

3rd DAG (pseudotumor)

- X (exposure of interest) – pseudotumor
- Outcome – re-revision

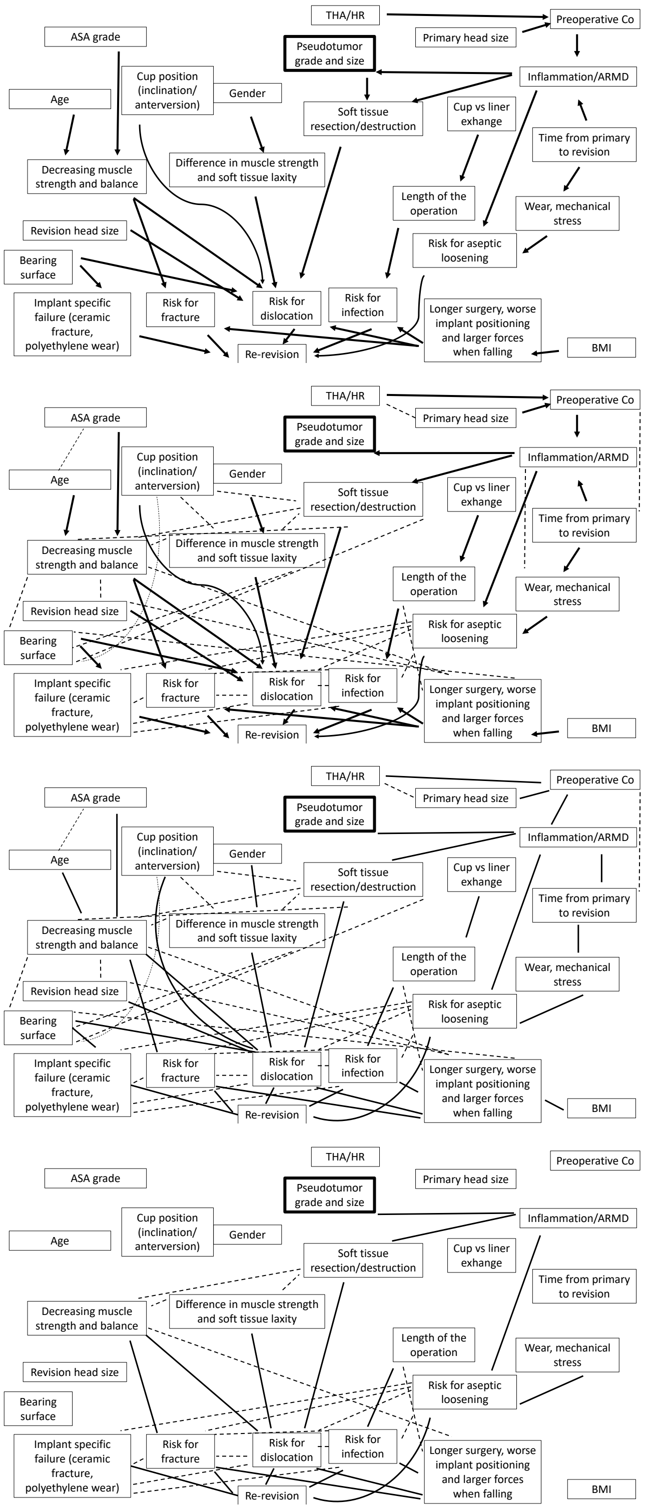

3rd DAG (pseudotumor)

- X (pseudotumor) is not dissociated from outcome. Variables affecting box "inflammation/ARMD" which connects X to outcome (THA/HR, primary head size, pre-revision Co, time from primary to revision) were excluded from the model

4th DAG (type of revision)

- X (exposure of interest) – type of revision (liner exchange, cup revision, resurfacing revision)
- Outcome – re-revision

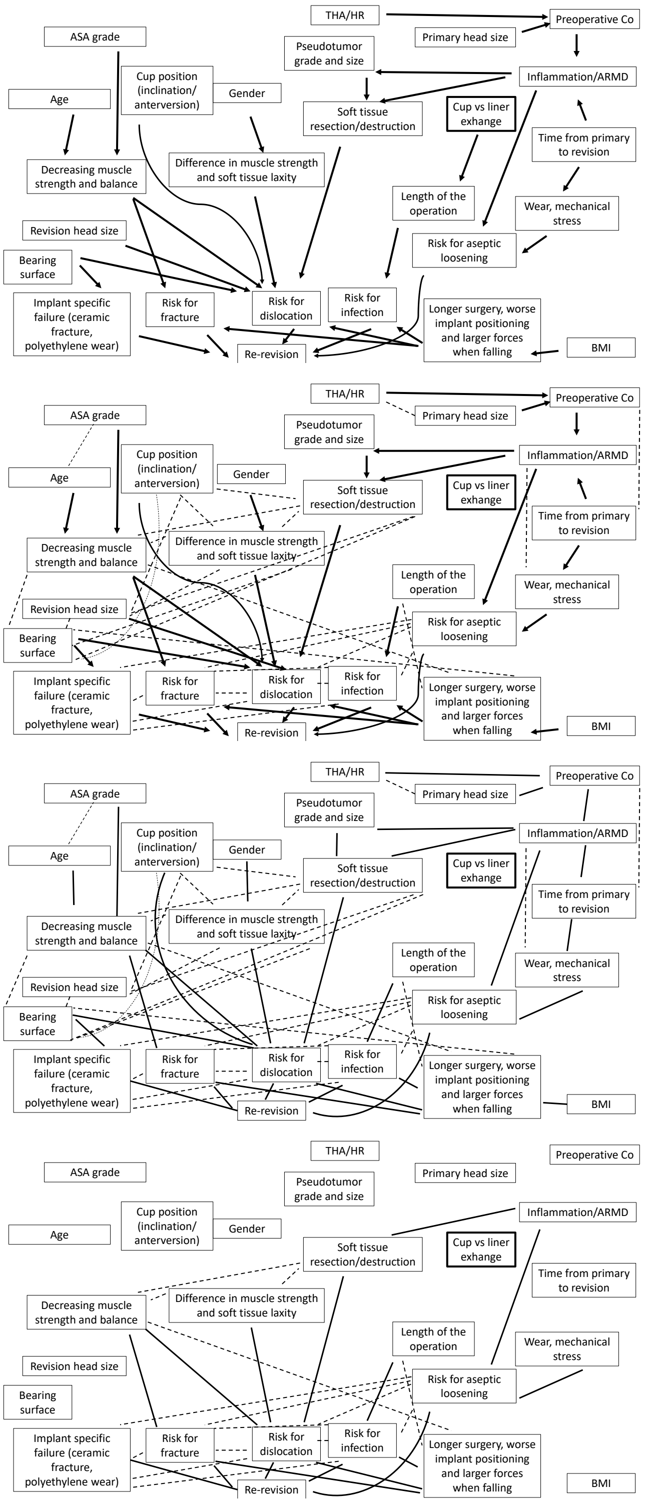

4th DAG (type of revision)

- X (type of revision) is dissociated from the outcome – all chosen covariates included

5th DAG (pre-revision cobalt)
- X (exposure of interest) – preoperative blood cobalt concentration
- Outcome – re-revision

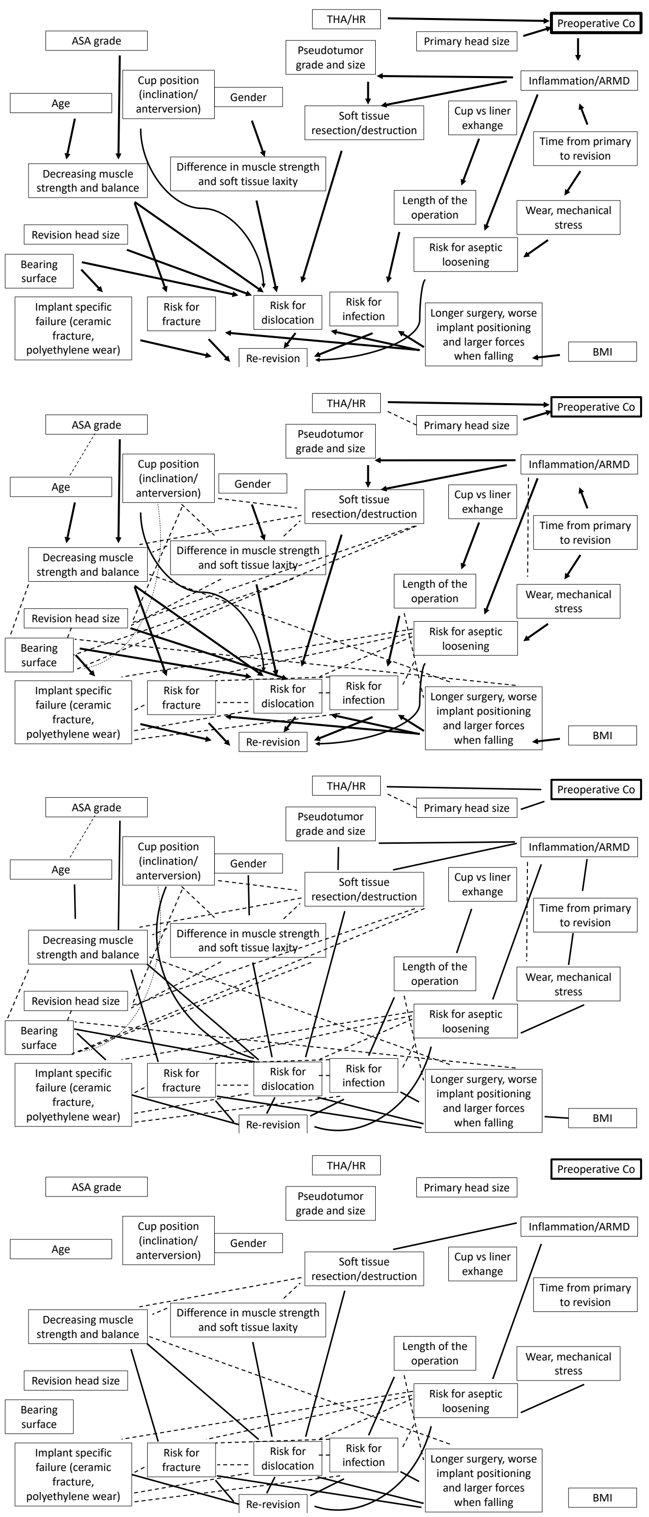

5th DAG (pre-revision cobalt)
- Pseudotumor is left out of the model because it is a descendant of preoperative Co
- X (preoperative Co) is dissociated from the outcome – all other covariates but pseudotumor included

6th DAG (revision head size)
- X (exposure of interest) – revision head size
- Outcome – dislocation

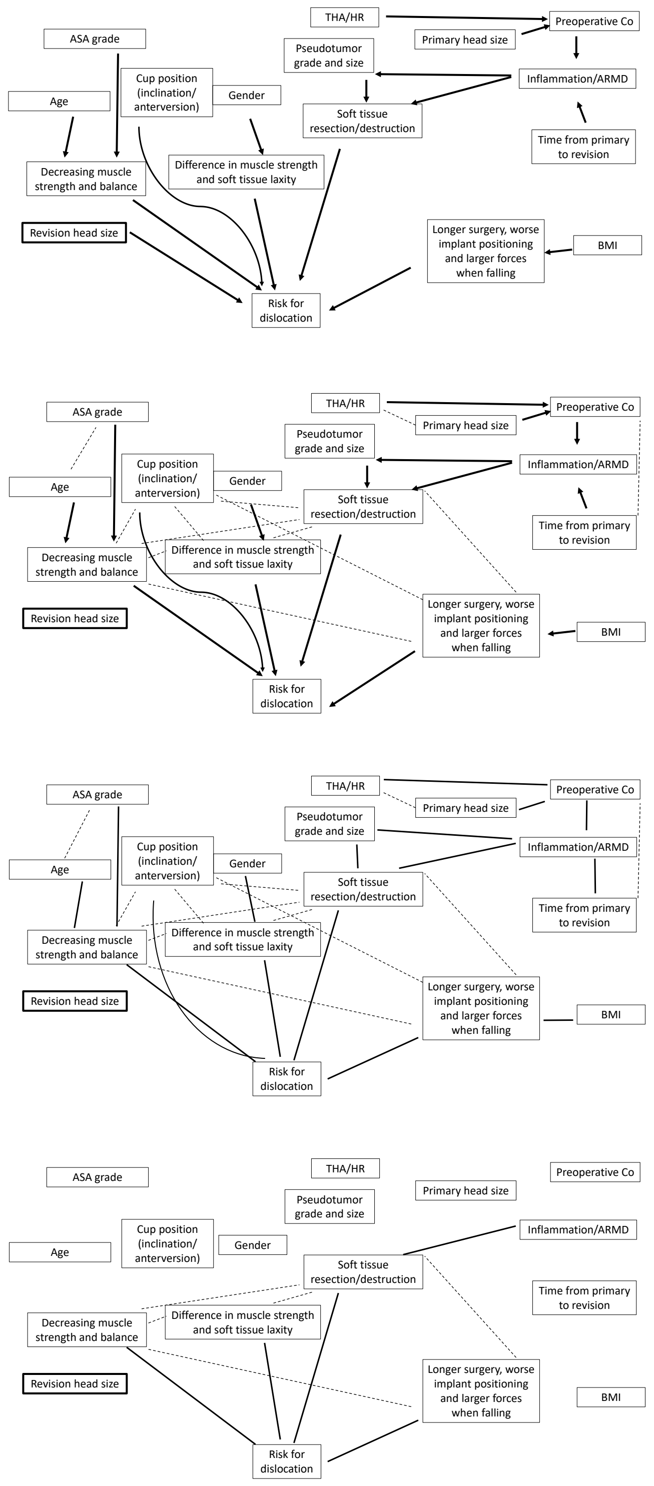

6th DAG (revision head size)
- X (revision head size) is dissociated from the outcome – all chosen covariates included

7th DAG (pseudotumor)
- X (exposure of interest) – pseudotumor
- Outcome – dislocation

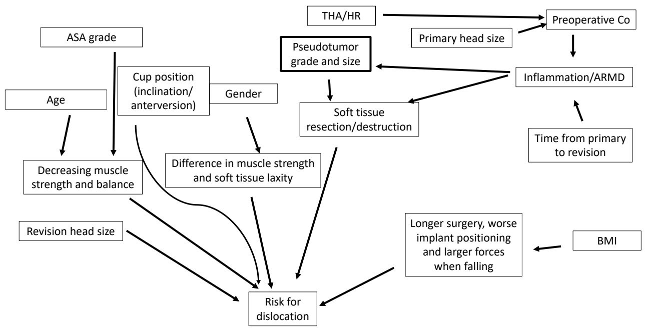

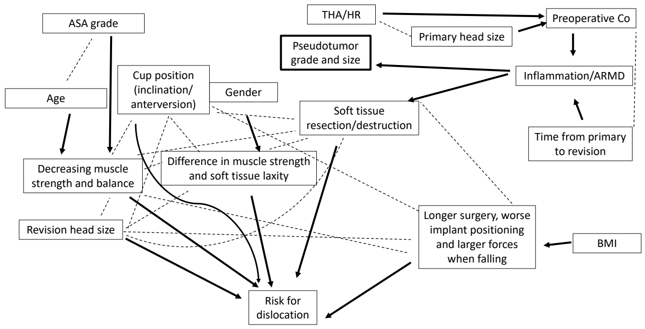

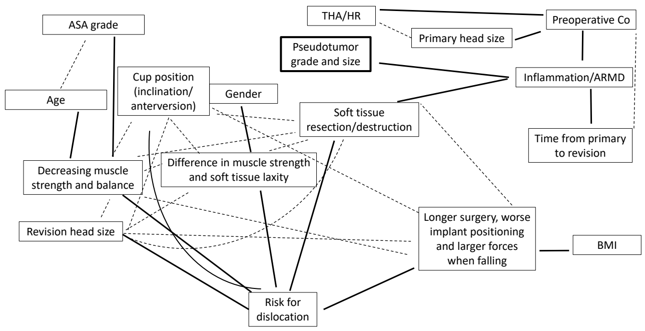

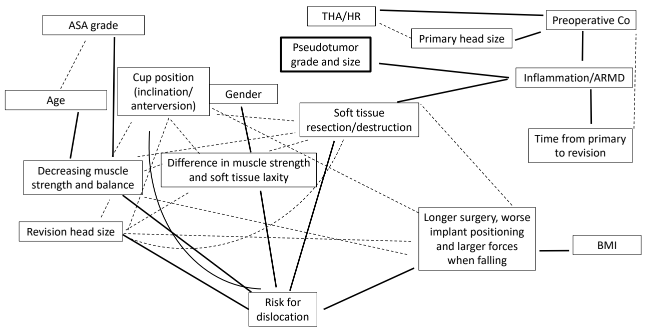

7th DAG (pseudotumor)
- X (pseudotumor) is not dissociated from outcome. Variables affecting box "inflammation /ARMD" which connects X to outcome (THA/HR, primary head size, pre-revision Co, time from primary to revision) were excluded from the model

8th DAG (pre-revision cobalt)
- X (exposure of interest) – preoperativen Co
- Outcome – dislocation

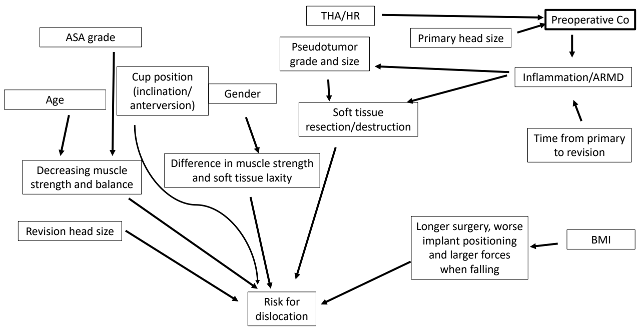

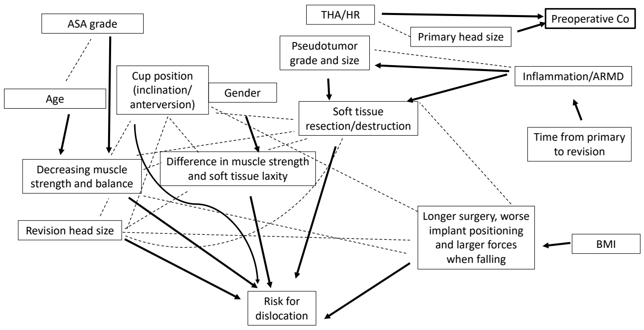

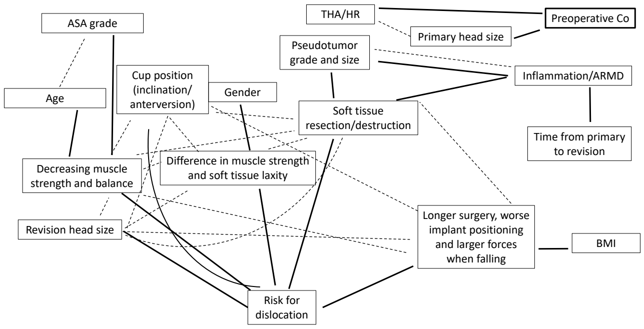

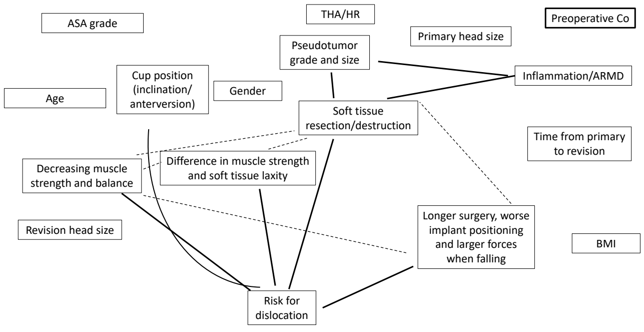

8th DAG (pre-revision cobalt)
- Pseudotumor is left out of the model because it is a descendant of preoperative Co
- X (preoperative Co) is dissociated from the outcome – all other covariates but pseudotumor included
